# Global Mutational Sweep of SARS-CoV-2: From Chaos to Order

**DOI:** 10.3389/fmicb.2022.820919

**Published:** 2022-02-08

**Authors:** Xin Wang, Mingda Hu, Yuan Jin, Boqian Wang, Yunxiang Zhao, Long Liang, Junjie Yue, Hongguang Ren

**Affiliations:** Beijing Institute of Biotechnology, State Key Laboratory of Pathogen and Biosecurity, Academy of Military Medical Sciences (AMMS), Beijing, China

**Keywords:** SARS-CoV-2, mutation, variant, trends, epidemic

The coronavirus disease 2019 (COVID-19) pandemic, caused by severe acute respiratory syndrome coronavirus 2 (SARS-CoV-2) (Zhou et al., 2020), has been ongoing for more than a year and a half. It has caused over 250 million infections with at least 5 million deaths worldwide as of November 2021. Meanwhile, millions of genome sequences of SARS-CoV-2 have been identified and shared globally. Various mutations have accumulated in the genome of the causative agent since its first identification, resulting in diverse variants of SARS-CoV-2 (Harvey et al., 2021). As a newly host-jumping virus to human beings, it is almost impossible to predict future new mutations or variants of SARS-CoV-2. However, evolutionary trends can be abstracted from the mutational pattern of large-scale genomes of SARS-CoV-2.

Based on 2,487,499 high-quality SARS-CoV-2 complete genome sequences (see Supplementary Materials) from GISAID Website (Shu and McCauley, 2017), we calculated the nucleotide mutation of each genome in comparison with Wuhan-Hu-1 (GenBank accession number NC_045512). We use the weekly mutation spectrum of the genomes to depict each country and compare their similarities within each 2-week window from Feb 24, 2020 to Aug 16, 2021. The similarities are calculated based on both the Cosine similarity (with windows flattened into row vectors) and the Frobenius similarity (the minus Frobenius norm of the matrix difference, see Supplementary Materials). Heat maps of these similarities were generated, along with the stack plots of the proportion of sequences that fall into defined variant groups. Interestingly, although the heat map and the stack plots were generated separately by mutational similarities and infected proportions, a convergent phenomenon was observed between them in the figure (Figure 1).

**Figure 1:**
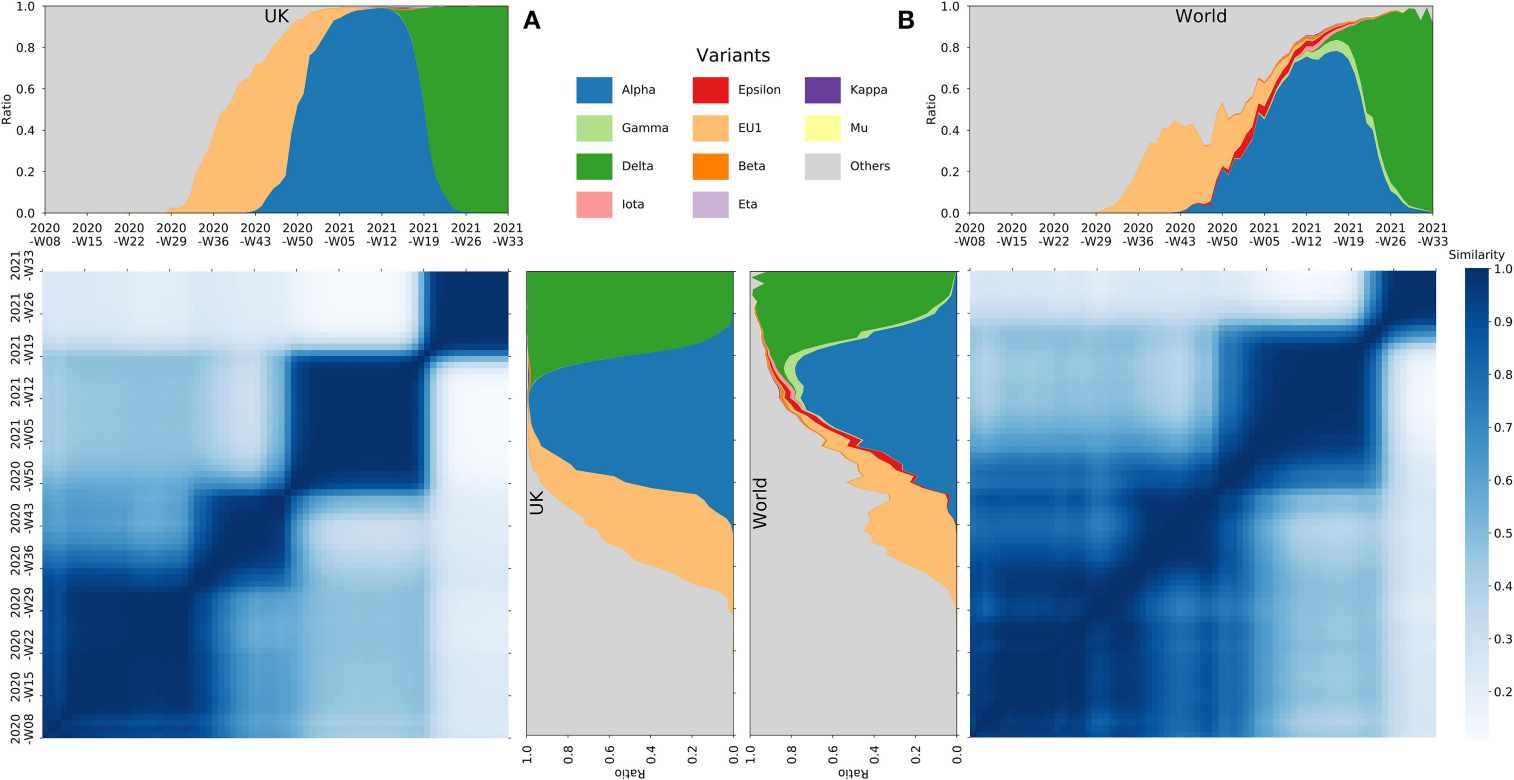
The Cosine similarity of the mutational spectrum of the SARS-CoV-2 genomes within the UK **(A)** and the whole world **(B)**, along with the stack plots of the proportion of the number of sequences, over time, that fall into defined variant groups.

It has been reported recently that the evolution of SARS-CoV-2 might have been undergoing selective sweeps (Kang et al., 2021). While from our observations, the mutation of SARS-CoV-2 genomes has evolved from an early chaotic phase to a state dominated by certain variants. The mutational heat map clearly demonstrates successive dark squares (see Figure 1 and Supplementary Figures 1–10), with each indicating relatively similar and stabilized mutation spectrums during that time period. Each square represents a specific phase during the pandemic, which synchronizes well with the contemporaneous predominant variant in the stack plot. From one phase to the next, the mutation of SARS-CoV-2 undergoes significant sweeps, in which previous variants (mutation combinations) are swept and replaced by new ones with possible adaptive advantages. Over time, the replacing process for later sweeps may have been accelerated, which can be seen from the sharp borders of later squares in the figure. Furthermore, later squares are darker than earlier ones, suggesting an increasingly genomic homogeneity over phases, which is due to more purified sweeps of variants as the pandemic goes on. The driving forces behind this phenomenon may be related to the enhanced fitness or adaptation of variants to human beings.

We have examined the aforementioned observation in a number of countries with large-scale SARS-CoV-2 sequences separately. Despite the regional differences, the conclusion holds for almost all situations (see Supplementary Figures 1–10), which all show phases (squares) divided by mutational sweeps. Benefit from abundant genome sequences, the figure of the United Kingdom is considerably representative (see Figure 1A), which shows several clear phases divided by sweeps dominated by typical SARS-CoV-2 variants.

We further compared the mutation spectrums among different regions. Due to the difference in both the control measure and the prevalence of variants, the heat map shows a variety of shapes. Taking comparing the UK and the US as an example (see Supplementary Figures 11, 12), the persistence of *Alpha* variant and *Delta* variant in the UK are longer than those in the US, so the similar mutation spectrum shows rectangles rather than squares. Note the mutation spectrums of these two countries showed less similarities in 2020, indicating the early regional genomic difference between the two countries. Nevertheless, the emergence of *Alpha* and then *Delta* variants in 2021 quickly converged such regional diversity. This implies that the evolution of SARS-CoV-2 has been undergoing selective sweeps both regionally and globally, in which previous local predominant strains can be quickly replaced by imported variants, e.g., *Alpha* and *Delta* variants, which has evolutionary advantages either in transmission or host adaptation, or both.

At the time this manuscript being submitted, the *Delta* variant has almost completed its sweep process throughout the world and become a global dominant variant. New SARS-CoV-2 variants with enhanced fitness will surely emerge in the future to replace the former predominant variant, but the possibility of co-circulating of multiple competing variants is low. It seems that the SARS-CoV-2, after the host-jumping event, has finished the early stages in adaptation to human beings through chaotic mutations and evolved into relatively persistent stabilized adaptations. More or less like the seasonal influenza virus (Petersen et al., 2020), the alternation of epidemic strains of SARS-CoV-2 may become periodic. During the review process of this manuscript, a new SARS-CoV-2 variant named *Omicron* emerged, which has drawn much attention throughout the world. To date, it is widely accepted that the *Omicron* variant has an increased transmissibility with a shorter doubling time compared with the *Delta* variant (Salim and Quarraisha, 2021). The emergence of the *Omicron* variant and its ongoing quick replacing of the former *Delta* variant showed a high consistence with our observations and conclusions.

Altogether, our study demonstrates that the SARS-CoV-2 has evolved from early chaotic mutations into relatively persistent stabilized adaptations. This ongoing adaptation presents successive phases of the pandemic, along with stronger sweeps and increasingly global homogeneity driven by the continuous emergence of SARS-CoV-2 variants. The completion of stage transition of COVID-19 might substantially prolong the pandemic with repeating epidemics, making it vital to strengthen the surveillance of SARS-CoV-2 globally. Meanwhile, vaccine development and vaccination strategies may should be updated accordingly.

## Author Contributions

HR and JY formulated the study. XW and MH performed the research and analyzed the data. YJ, BW, YZ, and LL participated in analysis and discussion. XW and HR drafted the manuscript. All authors contributed to the article and approved the submitted version.

## Funding

This work was supported by the National Natural Science Foundation of China (Grant Nos. 31800136, 32070025, and 82041019).

## Conflict of Interest

The authors declare that the research was conducted in the absence of any commercial or financial relationships that could be construed as a potential conflict of interest.

## Publisher's Note

All claims expressed in this article are solely those of the authors and do not necessarily represent those of their affiliated organizations, or those of the publisher, the editors and the reviewers. Any product that may be evaluated in this article, or claim that may be made by its manufacturer, is not guaranteed or endorsed by the publisher.
