# Supplementary material for: Global Mutational Sweep of SARS-CoV-2: From Chaos to Order
Source: Front Microbiol. 2022 Feb 8;13:820919. doi: 10.3389/fmicb.2022.820919 (PMC8861355; doi:10.3389/fmicb.2022.820919)
Supplement: Supplementary file 1 [file Data_Sheet_1.PDF]

Appendix

Table of Contents

Materials and Methods **2**
Supplementary Figure S1 .... **3**
Supplementary Figure S2 .... **4**
Supplementary Figure S3 .... **5**
Supplementary Figure S4 .... **6**
Supplementary Figure S5 .... **7**
Supplementary Figure S6 .... **8**
Supplementary Figure S7 .... **9**
Supplementary Figure S8 .... **10**
Supplementary Figure S9 .... **11**
Supplementary Figure S10 .... **12**
Supplementary Figure S11 .... **13**
Supplementary Figure S12 .... **14**
Genome Sequence Availability **15**

Materials and Methods

We collected 2,487,499 high-quality SARS-CoV-2 complete genome sequences from GISAID Website (c.f. Fasta ID.csv for detailed information). For each genome, the nucleotide mutation is calculated in comparison with Wuhan-Hu-1 (GenBank accession number NC_045512). We study the mutation spectrum of genomes in a given region, focusing on the whole world and four object countries, Brazil, India, the United Kingdom, and the United States. Nucleotide mutations with global occurrence of less than 10,000 are considered infrequent and then abandoned. Additionally, mutations absent in any of the four countries are neither included. Thus, 475 major nucleotide mutations remain for further studies.

We depict each region by its weekly mutation spectrum of genomes, consisting of the weekly proportion of 475 major mutations from Feb 24, 2020, to Aug 16, 2021, namely a period of 78 weeks. A two-week window is screened in the spectrum and the similarity is calculated, using both the Cosine similarity and the Frobenius similarity.

For the Cosine similarity, the matrix of each window is flattened into a row vector, then the Cosine similarity between flattened vectors $\boldsymbol{a}$ and $\boldsymbol{b}$ is calculated as follows.

$$similarity = \boldsymbol{a} \cdot \boldsymbol{b} \Big/ \sqrt{\sum_{i=1}^{n} {a_i}^2} \cdot \sqrt{\sum_{i=1}^{n} {b_i}^2}$$

For matrices of two windows, $\boldsymbol{A}, \boldsymbol{B} \in \mathbb{R}^{m \times n}$, we define the Frobenius similarity between windows by the minus Frobenius norm of the difference between matrices $\boldsymbol{A}$ and $\boldsymbol{B}$.

$$similarity = \|\boldsymbol{A} - \boldsymbol{B}\|_F = \sqrt{\sum_{i=1}^{m} \sum_{j=1}^{n} (A_{ij} - B_{ij})^2}$$

Heat maps are generated from these similarities between spectrums. For each region, we also analyze the SARS-CoV-2 genome sequences and draw the stack plots of the proportion of defined variant groups. The correspondence between heat maps and contemporaneous variant groups in stack plots is conducive to our studies.

Supplementary Figure S1. The Cosine similarity of the mutational spectrum of the SARS-CoV-2 genomes within the whole world.

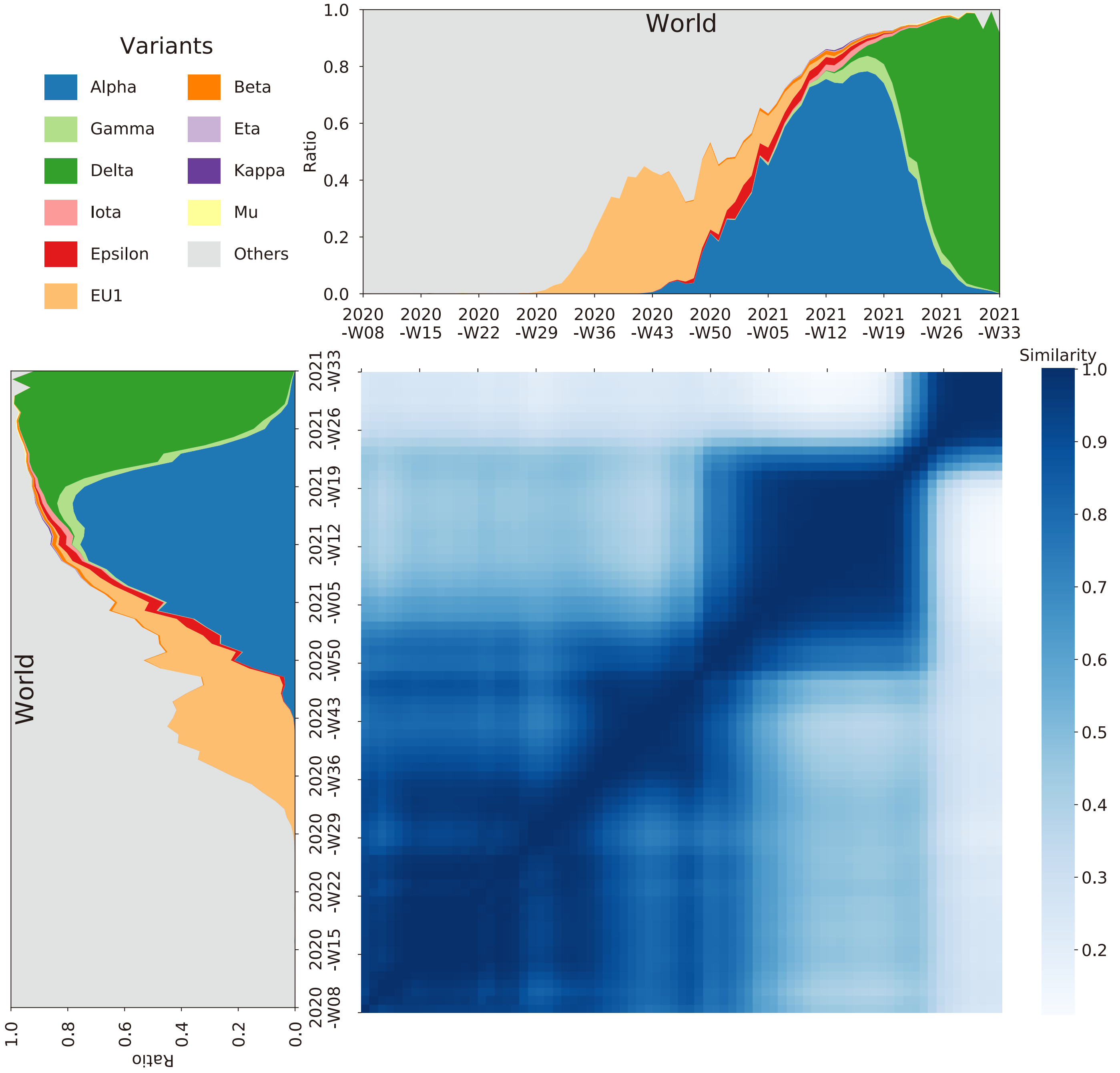

Supplementary Figure S2. The Frobenius similarity of the mutational spectrum of the SARS-CoV-2 genomes within the whole world.

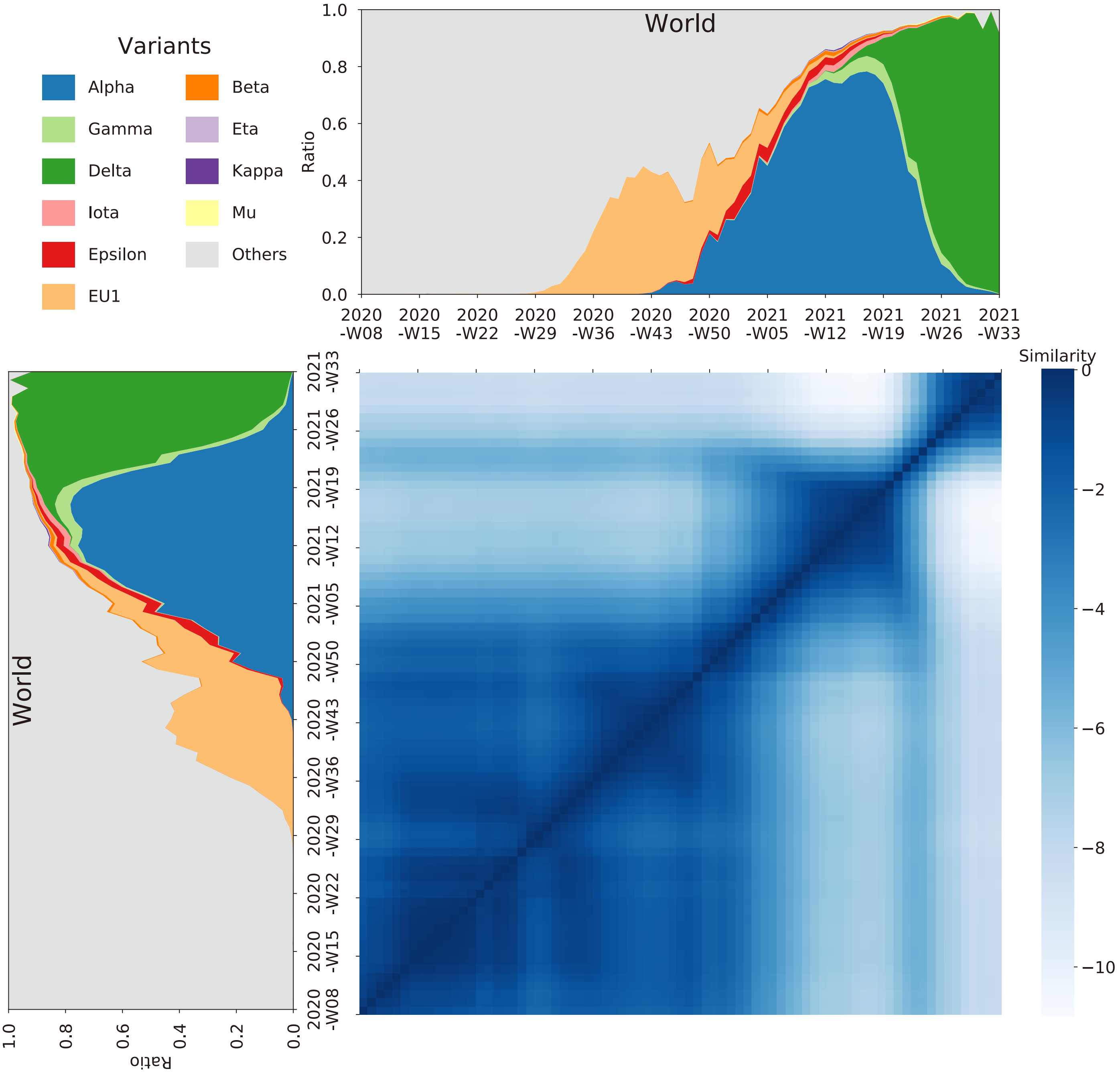

Supplementary Figure S3. The Cosine similarity of the mutational spectrum of the SARS-CoV-2 genomes within the UK.

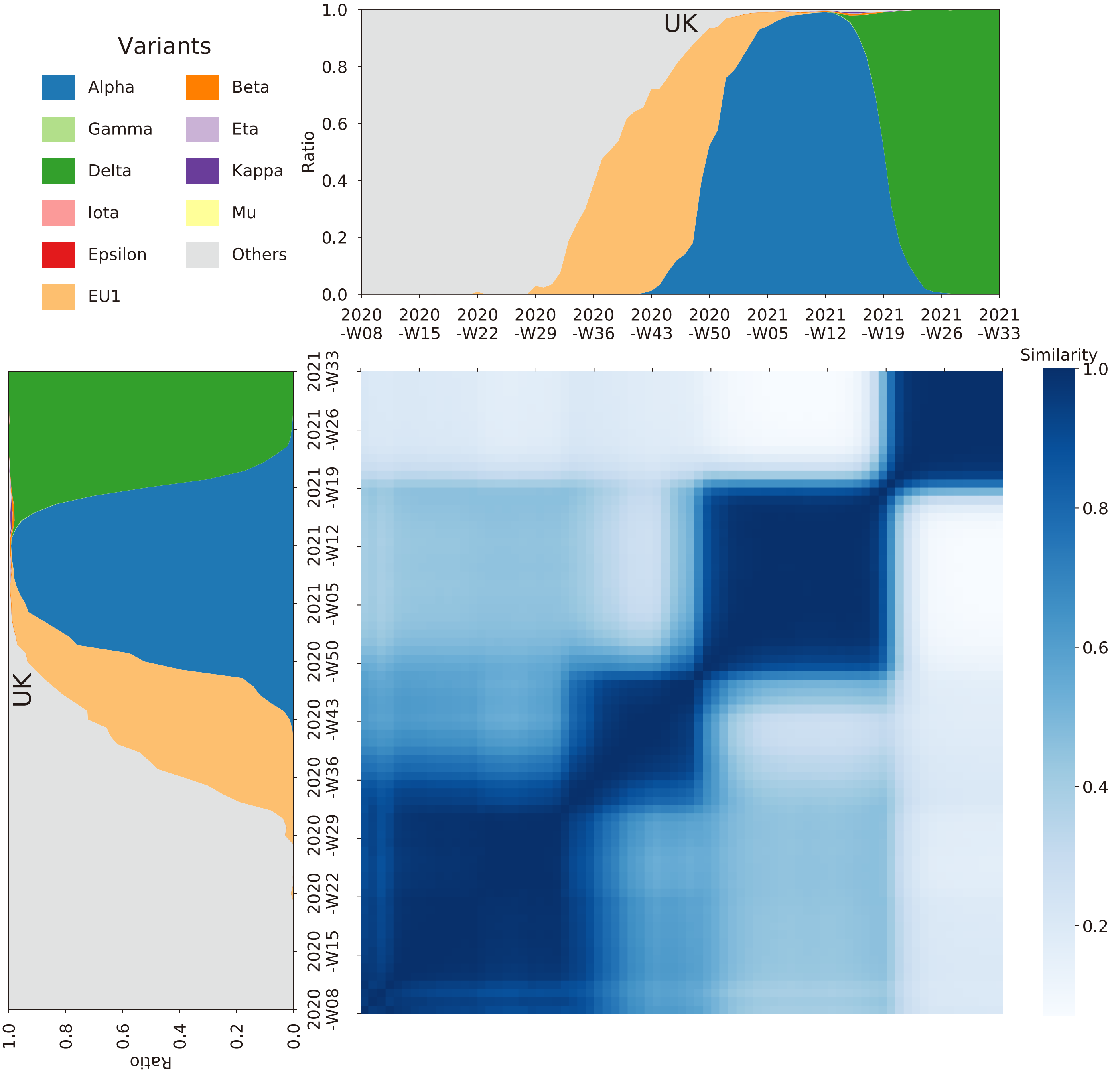

Supplementary Figure S4. The Frobenius similarity of the mutational spectrum of the SARS-CoV-2 genomes within the UK.

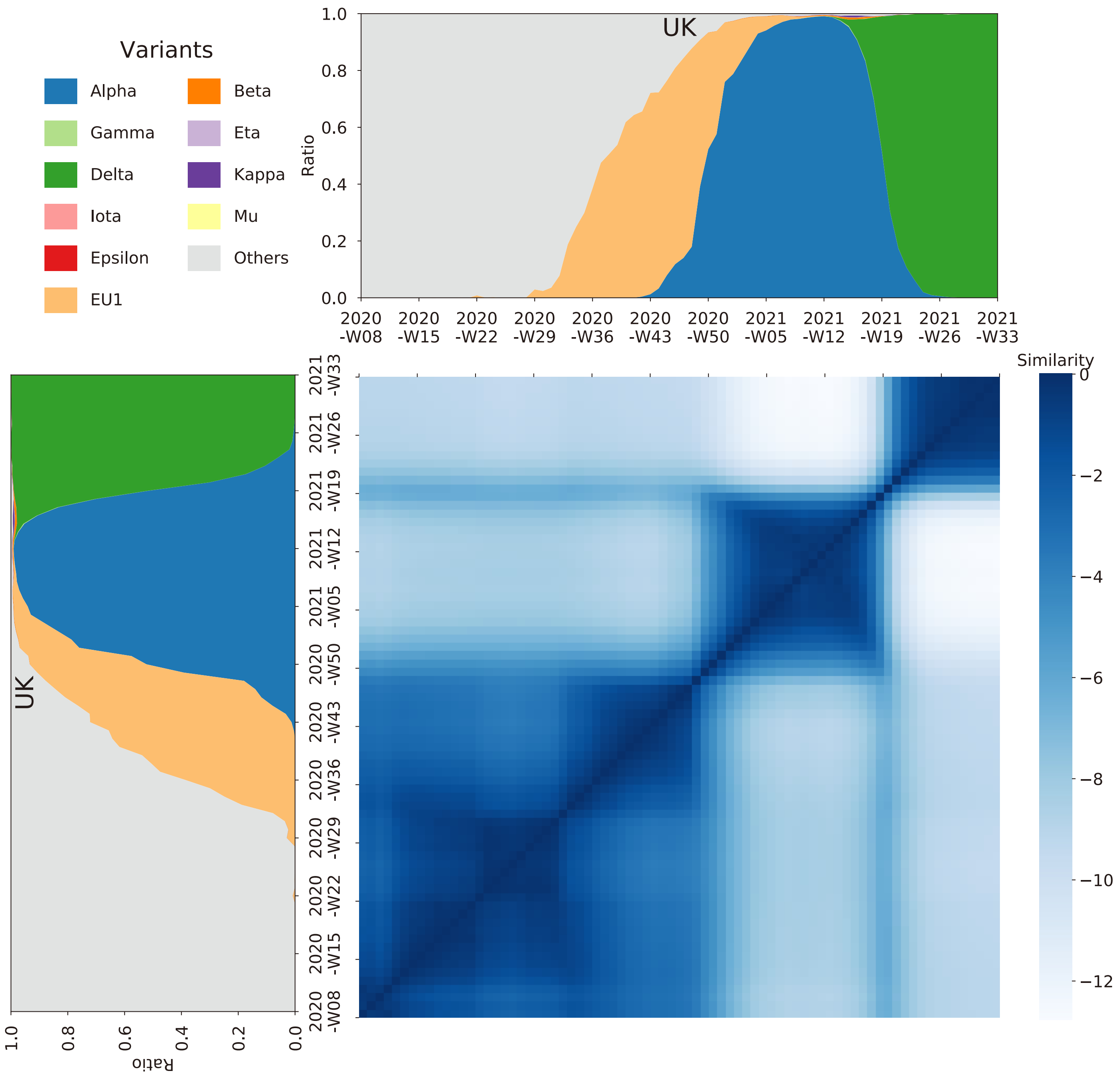

Supplementary Figure S5. The Cosine similarity of the mutational spectrum of the SARS-CoV-2 genomes within the US.

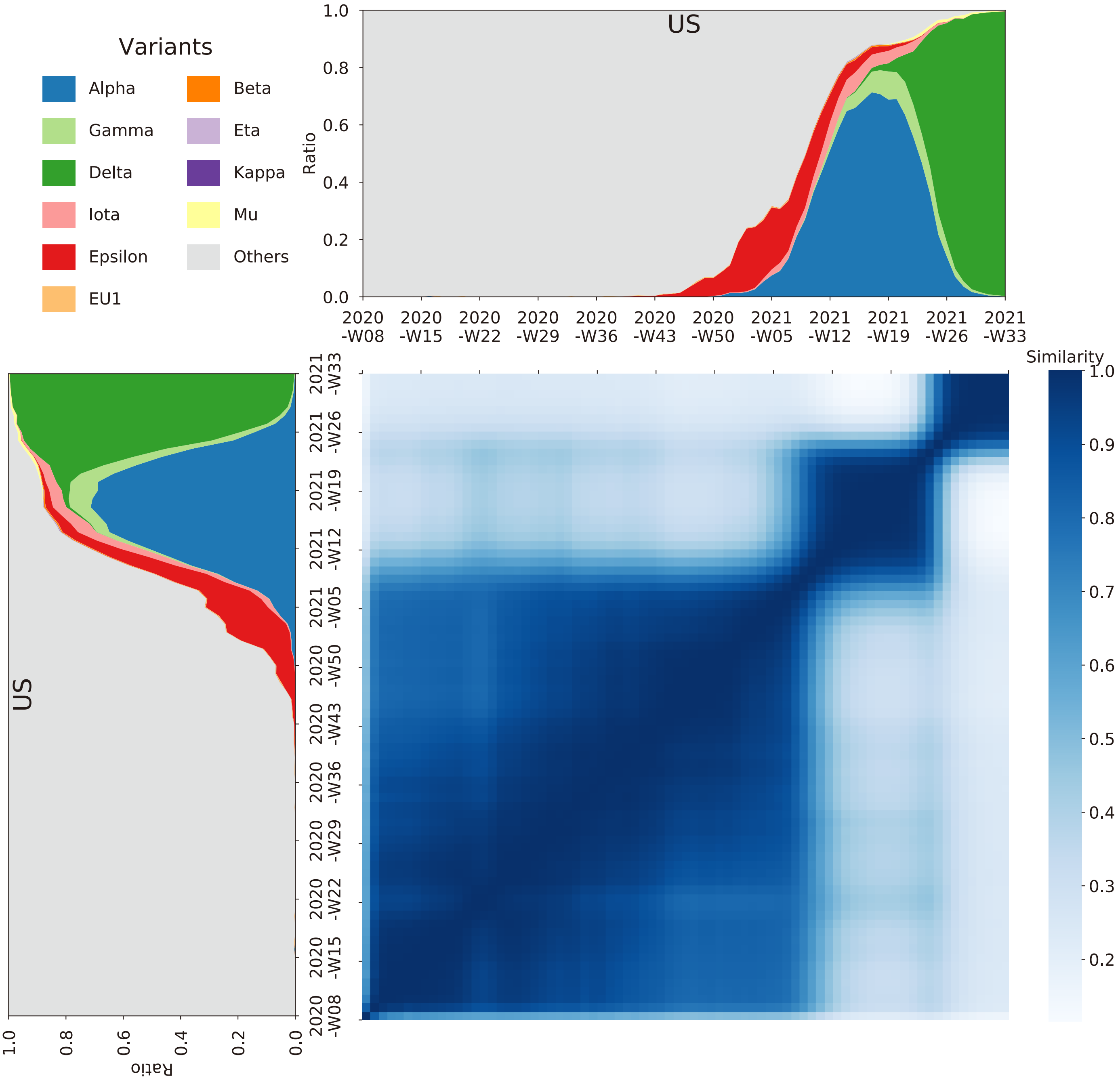

Supplementary Figure S6. The Frobenius similarity of the mutational spectrum of the SARS-CoV-2 genomes within the US.

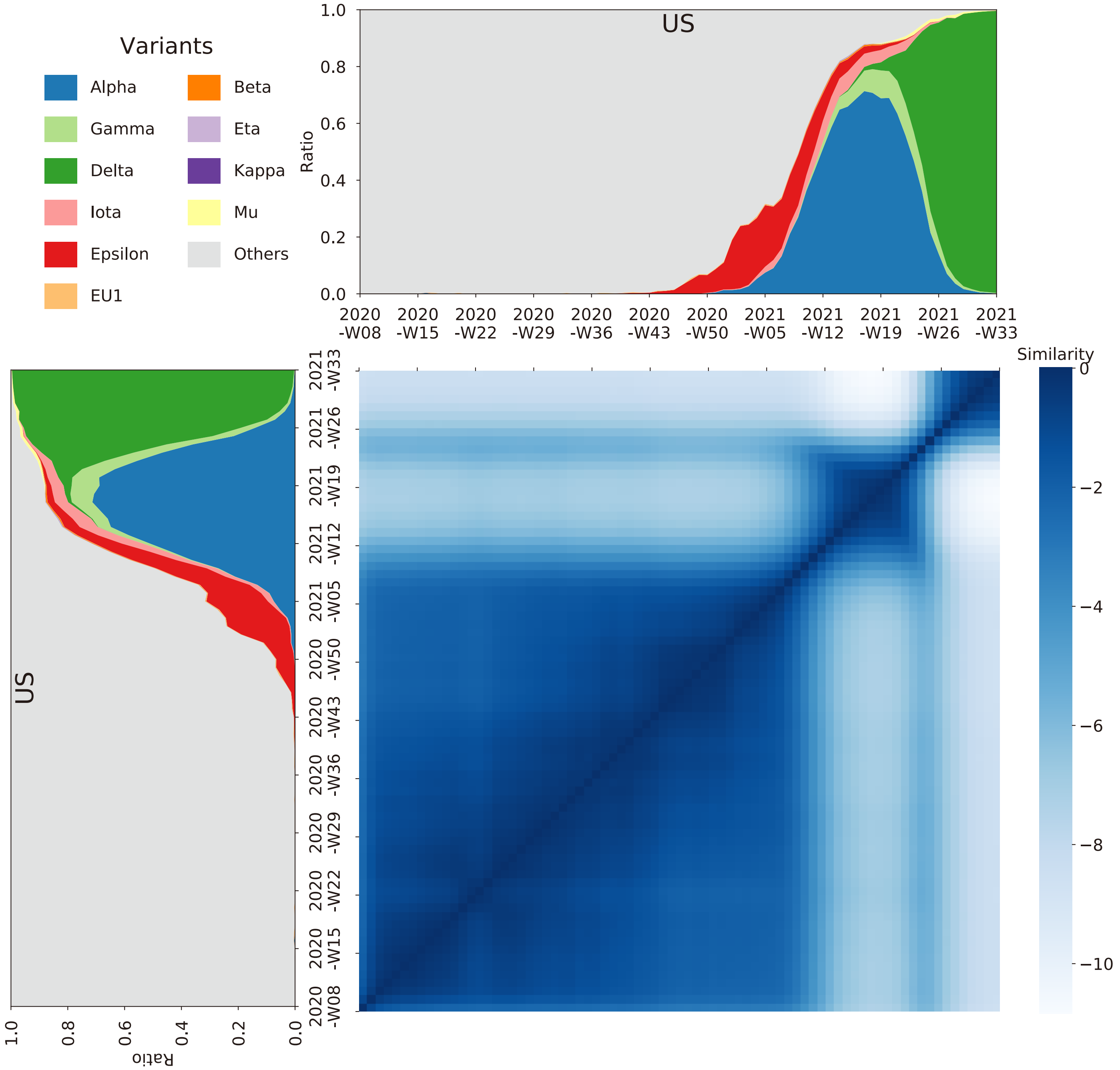

Supplementary Figure S7. The Cosine similarity of the mutational spectrum of the SARS-CoV-2 genomes within Brazil.

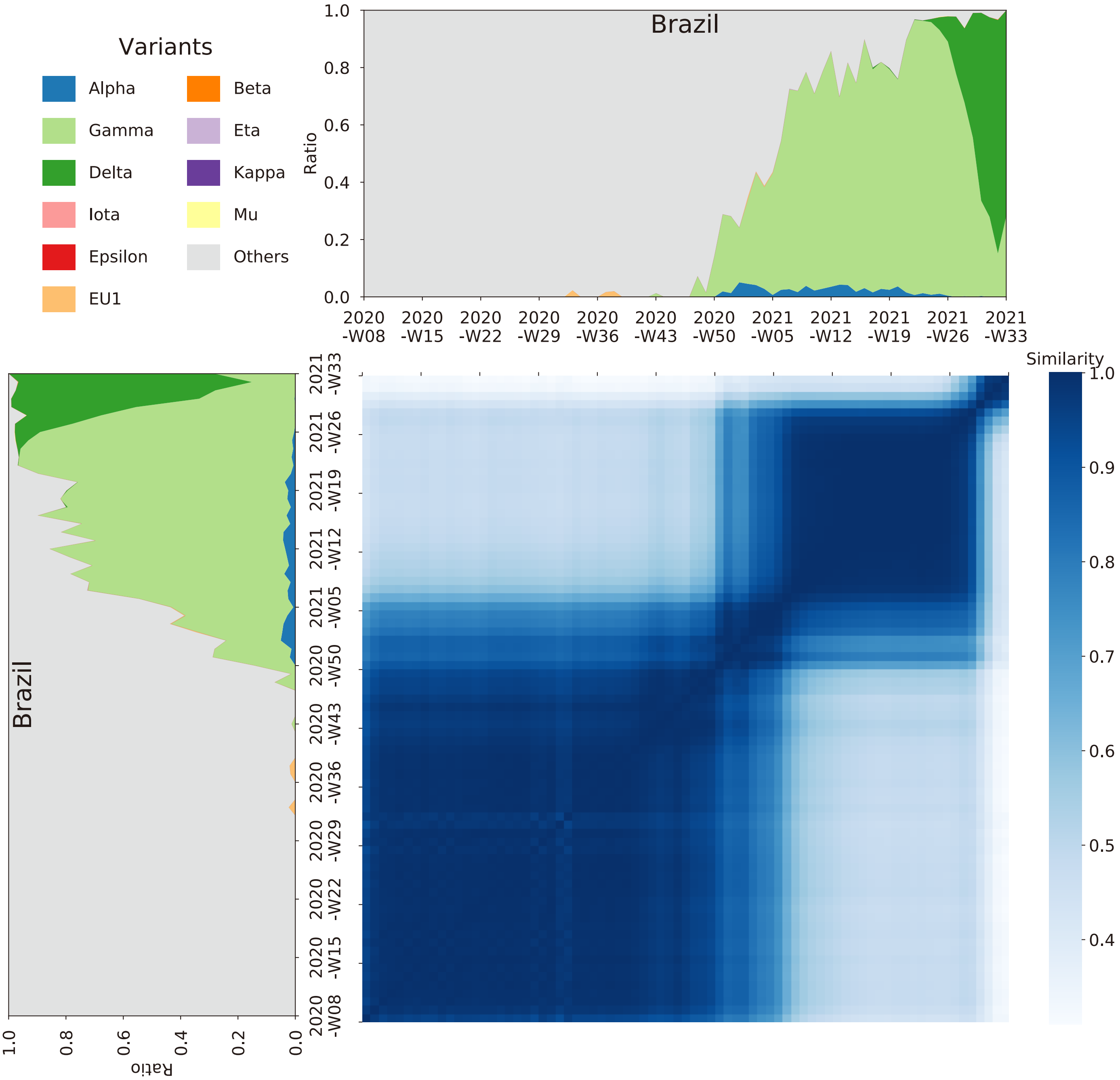

Supplementary Figure S8. The Frobenius similarity of the mutational spectrum of the SARS-CoV-2 genomes within Brazil.

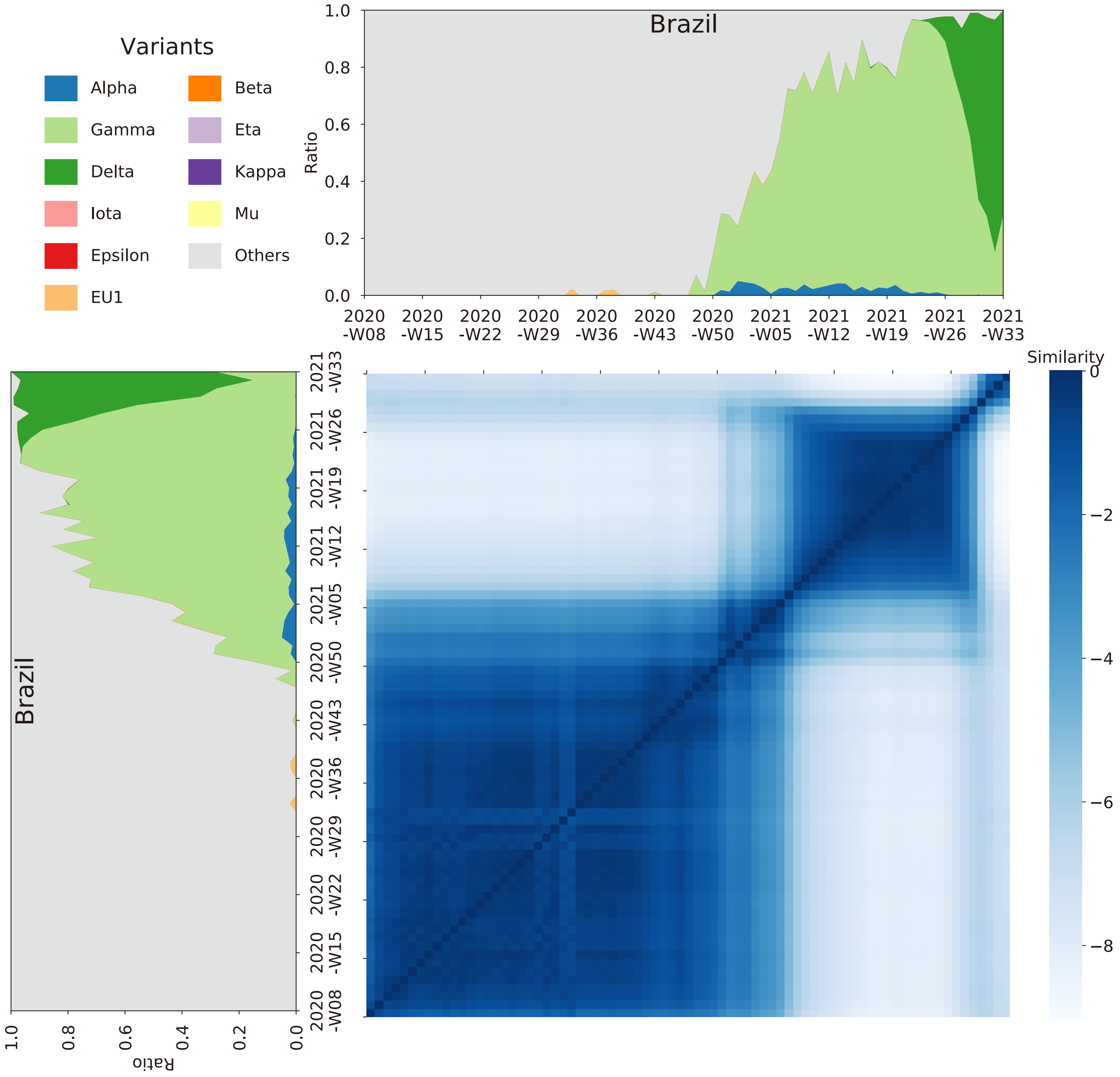

Supplementary Figure S9. The Cosine similarity of the mutational spectrum of the SARS-CoV-2 genomes within India.

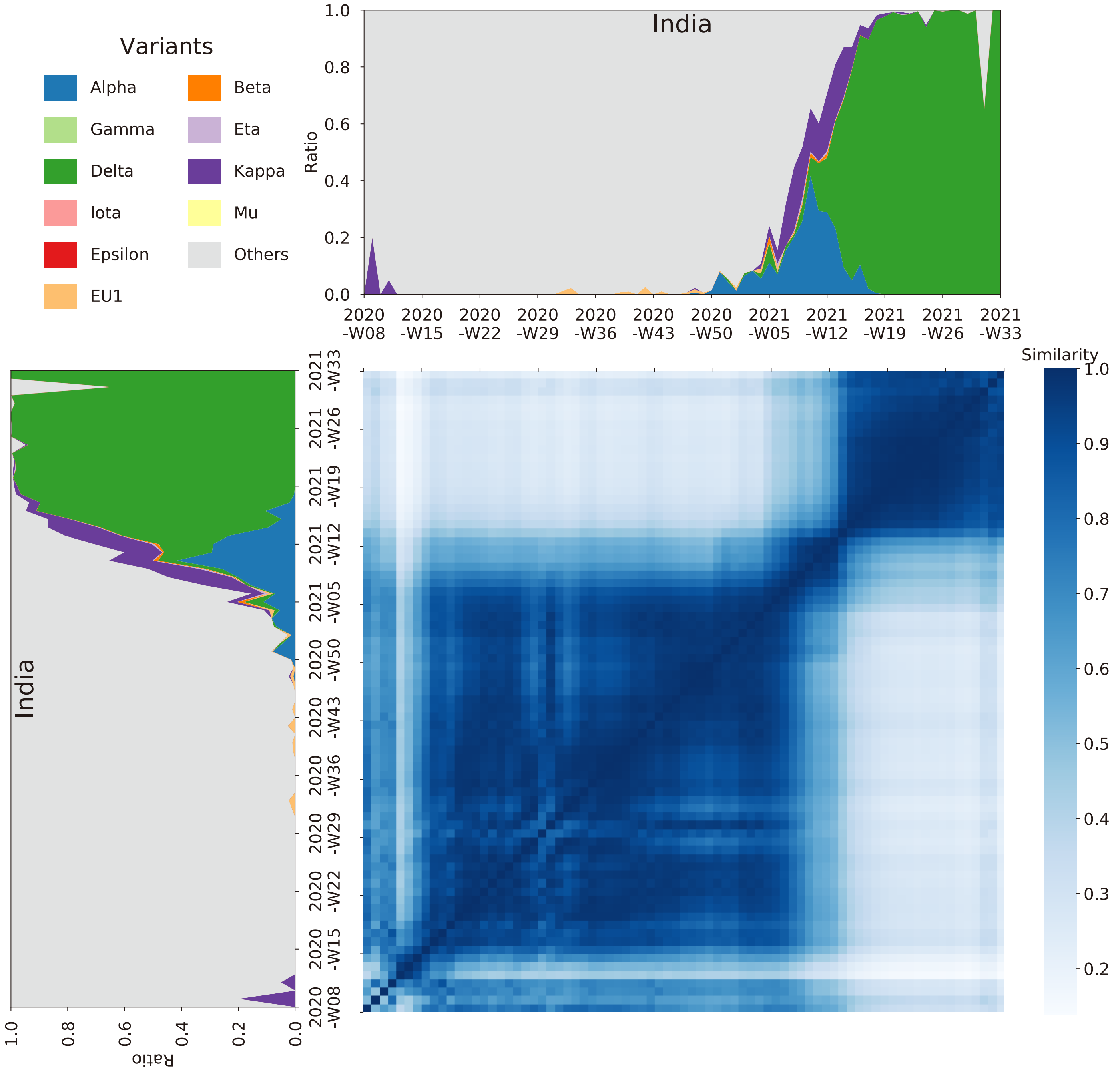

Supplementary Figure S10. The Frobenius similarity of the mutational spectrum of the SARS-CoV-2 genomes within India.

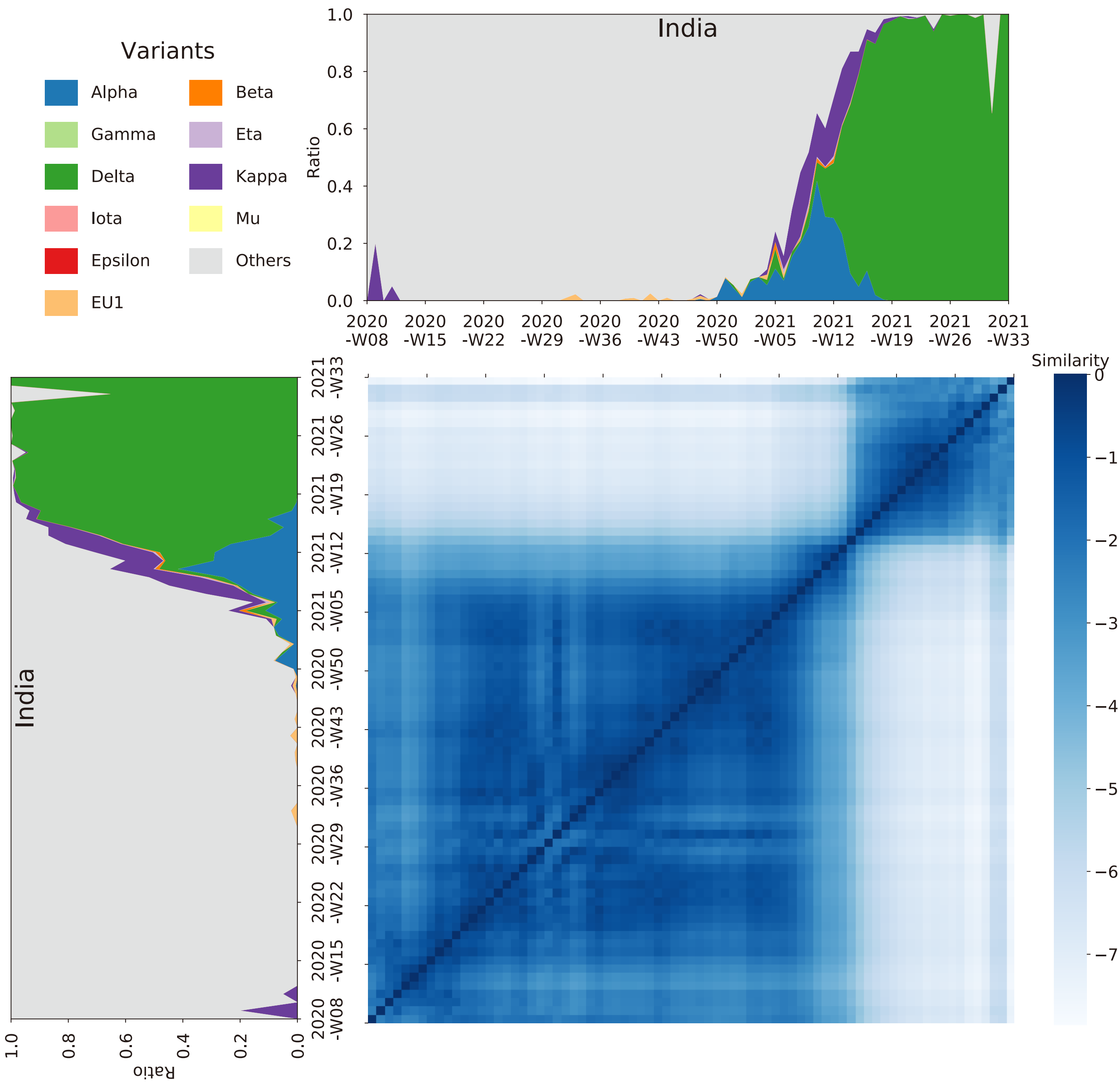

Supplementary Figure S11. The Cosine similarity of the mutational spectrum of the SARS-CoV-2 genomes between the UK and the US.

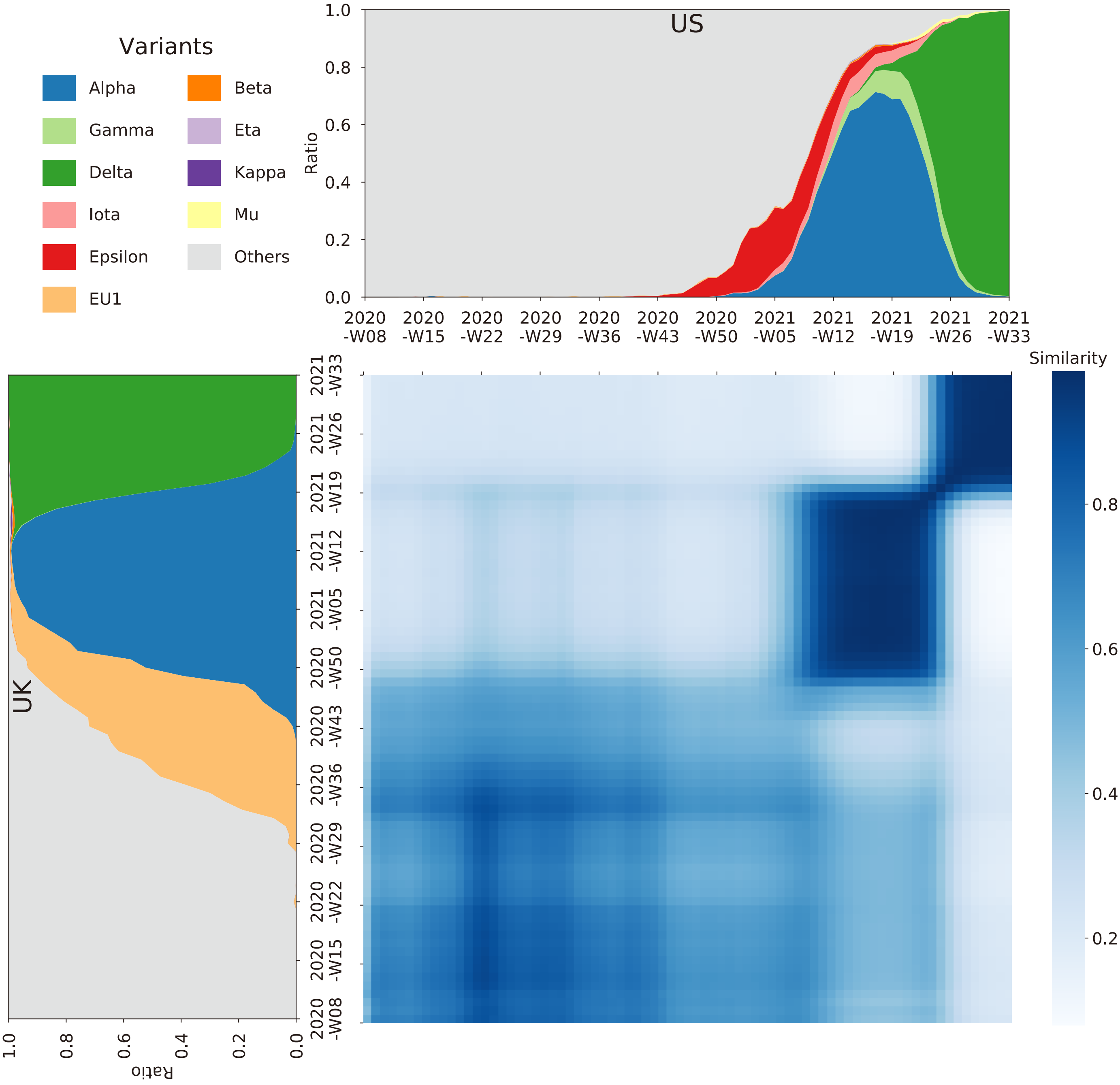

Supplementary Figure S12. The Frobenius similarity of the mutational spectrum of the SARS-CoV-2 genomes between the UK and the US.

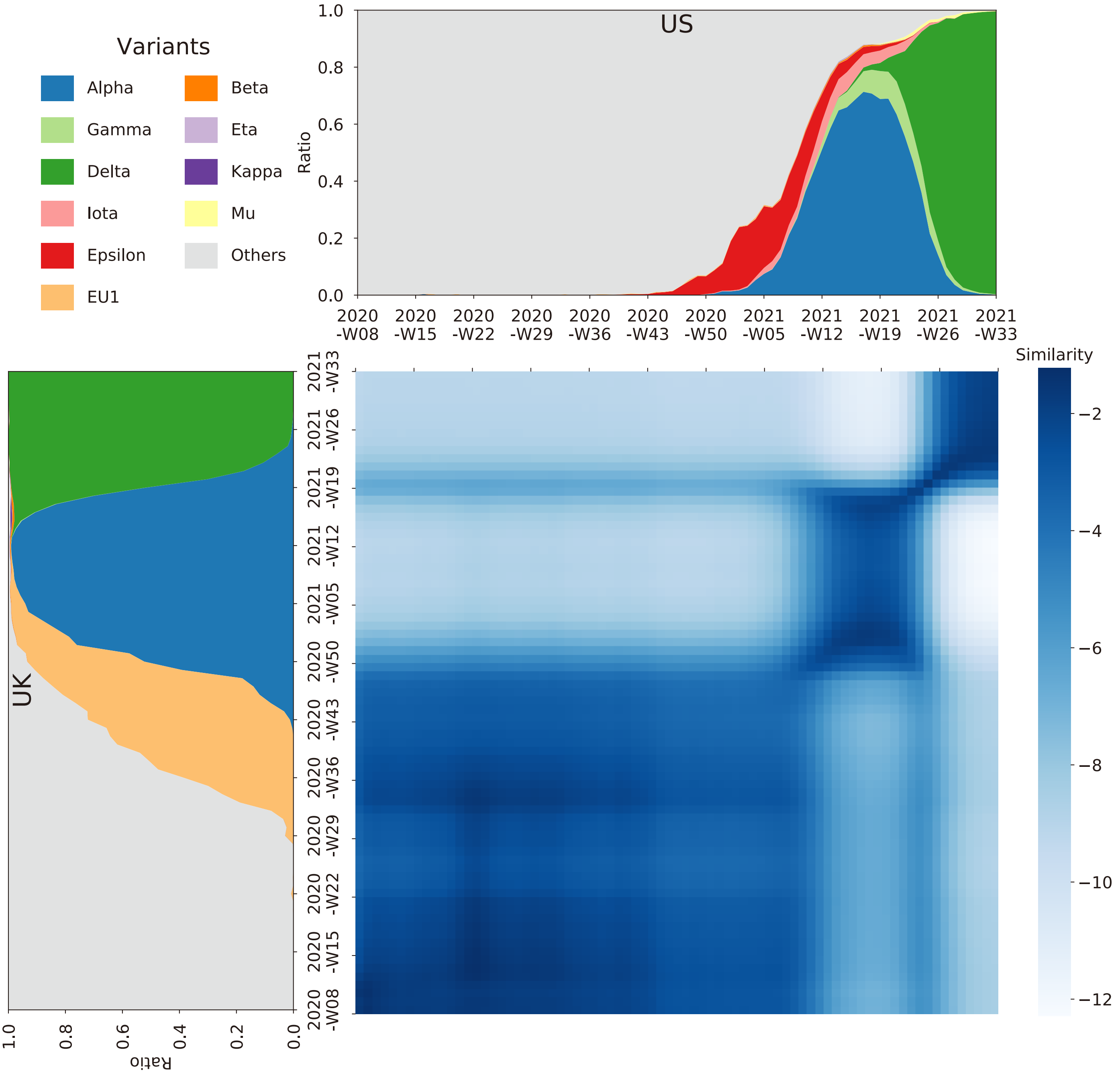

Genome Sequence Availability

We downloaded SARS-CoV-2 genome sequences as of Sep 08, 2020, from GISAID Website[1]. Only high-quality complete sequences are retained and thus we obtained 2,487,499 genome sequences of SARS-CoV-2. Please c.f. Supplementary_Fasta_ID.csv for detailed information of those genome sequences.

References

1. Shu Y and McCauley J. GISAID: Global initiative on sharing all influenza data - from vision to reality. *Euro Surveill*. 2017;**22**(13).
